# A biased coin up-and-down sequential allocation trial to determine the ED90 of intrathecal sufentanil combined with ropivacaine 2.5 mg for labor analgesia

**DOI:** 10.3389/fmed.2023.1275605

**Published:** 2024-01-08

**Authors:** Qiaoli Yin, Bin Yu, Hua Hao, Gang Li, Junyan Sun, Hao Kong, Liqin Deng

**Affiliations:** ^1^Department of Anesthesiology, General Hospital of Ningxia Medical University, Yinchuan, China; ^2^Department of Anesthesiology, Peking University First Hospital, Ningxia Women’s and Children’s Hospital, Yinchuan, China; ^3^Department of Anesthesiology, Guolong Hospital, Yinchuan, China; ^4^Department of Anesthesiology, Peking University First Hospital, Beijing, China

**Keywords:** sufentanil, intrathecal, ED90, labor analgesia, biased coin up-and-down

## Abstract

**Purpose:**

To determine the 90 percent effective dose (ED90) of intrathecal sufentanil combined with ropivacaine 2.5 mg for labor analgesia and observe its safety for parturients and neonates.

**Methods:**

We conducted a prospective, double-blind, biased coin up-and-down study. We injected a fixed 2.5 mg ropivacaine combined with a designated dose of sufentanil intrathecally to observe the labor analgesic effect. The initial dose of sufentanil was assigned 1.0 μg, and the remaining doses were assigned as per the biased coin up-and-down method. The criterion of successful response was defined as VAS ≤ 30 mm after intrathecal injection at 10 min. Safety was evaluated in terms of maternal and neonatal outcomes.

**Results:**

The ED90 dose of intrathecal sufentanil combined with ropivacaine 2.5 mg (0.1%, 2.5 mL) was 2.61 μg (95% CI, 2.44 to 2.70 μg) by isotonic regression. No respiratory depression, hypotension, or motor block was observed. Thirty-one (77.5%) parturients complained of pruritus, and 14 (35.0%) suffered nausea and vomiting. Three neonates reported a 1 min Apgar score of ≤7, and none reported a 5 min Apgar score of ≤7.

**Conclusion:**

The ED90 of intrathecal sufentanil combined with ropivacaine 2.5 mg for labor analgesia was 2.61 μg. The dose is safe for parturients and neonates.

## 1 Introduction

Neuraxial analgesia is the most effective and prevailing way to provide labor pain relief. Epidural technique and combined spinal–epidural (CSE) technique are both recommended by guideline (1). Existing evidence suggests that compared with the epidural technique, the CSE technique demonstrated several potential advantages, including more rapid onset of analgesia, less need for analgesic rescue, lower incidence of urinary retention, and reduced rate of instrumental delivery (2). In the CSE technique, intrathecal injection of low-dose local anesthetics combined with lipophilic opioids can safely achieve adequate analgesia without motor block and offer rapid onset and high maternal satisfaction (3, 4).

Studies have explored the optimal dose of intrathecal sufentanil for labor analgesia. In the early years, Herman et al. (5) established the effective dose (ED) 50 and ED95 for intrathecal sufentanil alone in laboring parturients were 2.6 [95% confidence interval (CI), 1.8–3.2] and 8.9 (7.5–11.5) μg, respectively, when a successful response was determined as an absolute VAS ≤ 25 mm. Wong et al. (6) reported the optimal dose of intrathecal sufentanil in combination with 2.5 mg bupivacaine was 2.5 μg, which provided analgesia comparable to higher doses and a lower incidence of nausea and vomiting and less severe pruritus. In recent years, ropivacaine has been increasingly used in labor analgesia due to its properties of a better separation between sensory and motor block and a lower systemic toxicity than bupivacaine (7, 8). However, the optimum dose of sufentanil with ropivacine was not well clarified. We designed a biased coin up-and-down sequential allocation trial to determine the ED90 of intrathecal sufentanil combined with ropivacaine 2.5 mg for labor analgesia.

## 2 Materials and methods

### 2.1 Study design and ethics

We conducted a prospective, double-blind, sequential allocation trial. The research protocol was approved by the Research Ethics Committee at Peking University First Hospital Ningxia Women’s and Children’s Hospital in Yinchuan, China (KJ-LL-2021-42, approval date November 25, 2021). The study was registered in Chinese Clinical Trial at chictr.org.cn (identifier: ChiCTR2300068408). Written informed consent was obtained from all participants. Our study used the CONSORT reporting guidelines (9).

### 2.2 Patients

The inclusion criteria included: (i) age 18–35 years; (ii) American Society of Anesthesiologists (ASA) physical status I – II; (iii) gestational age ≥ 37 weeks; (iv) nulliparous women with singleton pregnancy; (v) cervical dilatation between 2 and 4 cm; (vi) cephalic presentation; and (vii) no head pelvic asymmetry. The exclusion criteria included: (i) participants with pregnancy-induced hypertension; (ii) any contraindication for spinal or/and epidural analgesia; (iii) body temperature ≥ 37.5°C; and (iv) allergy to local anesthetics or opioids. The dropout criteria were: (i) puncture failure; (ii) accidental dural puncture; (iii) unilateral block; (iv) epidural catheter unintentionally entered the intrathecal cavity or blood vessel; and (v) epidural catheter detachment or blocked during labor analgesia.

### 2.3 Management of labor analgesia

Baseline maternal heart rate, non-invasive blood pressure, oxygen saturation, and fetal heart rate were measured between two uterine contractions. Baseline maternal visual analog score (VAS) was recorded during uterine contraction (VAS 0–100 mm, where 0 = painless and 100 = unbearable severe pain).

An intravenous catheter was established, and 500 mL of 0.9% saline was started. The parturient was positioned in a lateral decubitus position and routinely sterilized. The epidural space was identified at L3-L4 interspace via the midline approach with an 18-G, 8-cm Tuohy epidural needle using a loss of resistance to saline technique. A needle-through-needle technique was performed using a 25-G, 12-cm Whitacre spinal needle placed into the shaft of the previously sited epidural needle with confirmation of free-flow cerebrospinal fluid. A designated dose of sufentanil combined with 0.1% ropivacaine 2.5 mg was injected into the intrathecal space. After administration, the spinal needle was pulled out. A 19-G multiport wire-reinforced epidural catheter was inserted 5 cm into the epidural space. Maternal VAS scores were assessed at 5 and 10 min after intrathecal injection. After negative aspiration for cerebrospinal fluid and blood, all parturients received a 1% lidocaine test dose of 3 mL. Patient-controlled epidural analgesia (PCEA) was initiated immediately after VAS assessment at 10 min with the following parameters: ropivacaine 1 mg/mL combined with sufentanil 0.5 μg/mL, background infusion at 6 mL/h, demand dose of 8 mL, lockout interval of 30 min, and hourly limit of 28 mL.

Maternal VAS scores, heart rate, non-invasive blood pressure, respiration, oxygen saturation, and fetal heart rate were monitored after labor analgesia. When maternal systolic blood pressure was <90 mm Hg, a dose of 6 mg ephedrine was administered; when maternal heart rate was <50 beats/min, a dose of 0.2–0.5 mg atropine was administered. Intrapartum fever was defined as maternal body temperature ≥ 38°C during labor analgesia (10). Urinary retention was defined as the implantation of a catheter or a disposable catheter when urine cannot be voided on its own during delivery (11). Motor block was assessed using a Modified Bromage Score (12). Four levels of maternal satisfaction assessment were graded as Very satisfied, fairly satisfied, not sufficiently satisfied, and not at all satisfied (13).

### 2.4 Biased-coin design up-down sequential method

Based on the biased coin up-and-down (BCUD) and our pilot study, the initial dose of sufentanil (Jiangsu Enhua Medicine Co, Ltd.) was set at 1.0 μg. Sufentanil dose for the subsequent subject was determined according to the responses of the previous subject using the BCUD with a possible increment or decrement of 0.25 μg. If the labor analgesia failed, the dose of sufentanil was increased by 0.25 μg in the subsequent parturient. If the labor analgesia succeeded, the next parturient would receive either the same dose (probability of 0.89) or a dose that was reduced by 0.25 μg (probability of 0.11).

The criteria used for determining a response were as follows: (i) successful labor analgesia: VAS ≤ 30 mm after intrathecal injection at 10 min; and (ii) failed labor analgesia: VAS > 30 mm after intrathecal injection at 10 min.

The biased coin up-and-down sequential allocation was carried out using a computer-generated list of random responses prepared by our statistician using Excel 2016 (Microsoft, Redmond, WA, USA). A research assistant used this list to provide the sufentanil dose for the next parturient. The anesthesiologists, nurses, and parturients remained blinded to the dose throughout the entire research process.

### 2.5 Endpoints of the study

Our primary endpoint was determining the ED90 of intrathecal sufentanil combined with ropivacaine 2.5 mg for labor analgesia. Our secondary outcomes were: (i) The visual analog scores at 5 min (T1), 10 min (T2), 15 min (T3), 30 min (T4), and 60 min (T5) after intrathecal injection, and at full cervical dilation (T6); (ii) Maternal adverse outcomes during labor analgesia, including pruritus, nausea and vomiting, urinary retention, respiratory depression, hypotension, intrapartum fever, and motor block, delivery mode, and abnormal fetal heart rate. (iii) Neonatal outcomes, including Apgar scores at 1 and 5 min after birth.

### 2.6 Sample size

The unknown distribution of data of the BCUD study prevents the development of rigorous rules to calculate the necessary sample size for the estimation of ED90. Pace et al. (14) suggested that including at least 20–40 patients will provide stable estimates of the target dose for the most realistic scenarios. We planned to enroll participants who met the inclusion and exclusion criteria and stopped when 40 patients had completed the study.

### 2.7 Statistical analysis

When ED90 is determined (*τ* = 0.9), the probability (B) = (1 − τ)/*τ* = (1–0.9)/0.9 = 0.1/0 0.9 ≈ 0.11, where B is the target probability percentage. If a failure is observed, the dose is always stepped up for the subsequent participant. If the dose is successful, the following patient received the next lower dose with a probability of B ≈ 0.11 (1/9) or the same dose with a probability of 1 − B = 0.89 (8/9). The success rate after adjusting the results is estimated by the Pooled Adjacent Violators Algorithm (PAVA).

The ED90 of sufentanil was calculated by isotonic regression, and the 95% (CI) was obtained with 2000 bootstrapped samples. Normal distribution data were presented as mean (standard deviation) and were compared using *t*-test between the two groups. Non-normal distribution data were presented as median (interquartile range) and were compared using Mann–Whitney *U* test. Categorical data were presented as number of patients (percentage) and were analyzed using the *χ*^2^ test. The statistical software used was R for Windows version 3.4.4 and SPSS for Windows version 24.0 (SPSS Inc., Chicago, Illinois).

## 3 Results

### 3.1 Participants statistical

**Figure 1:**
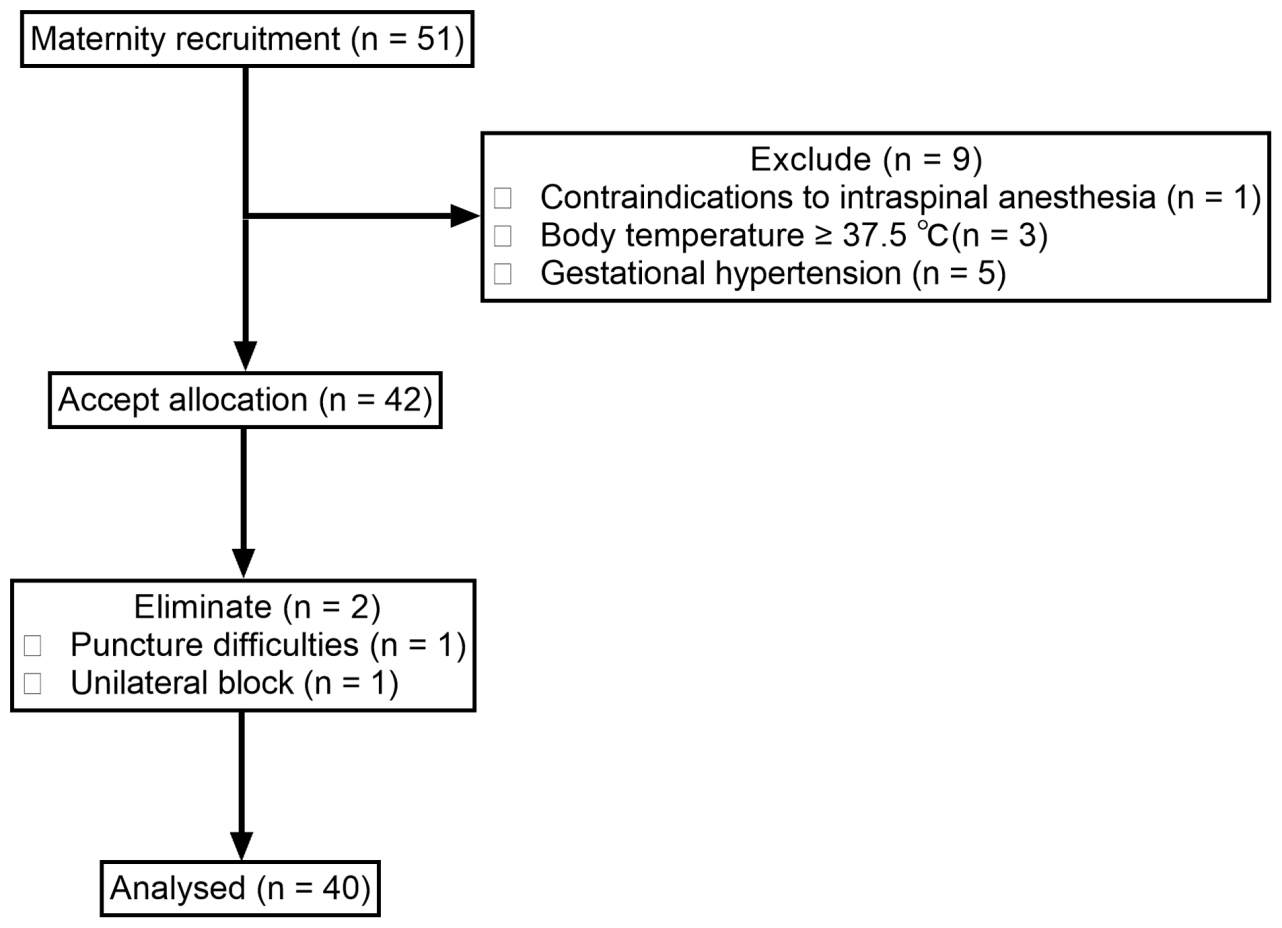
Flowchart of the study.

We screened 51 women who met the inclusion criteria from November 25, 2021, to December 31, 2022. Among them, one was excluded for contraindication to spinal or/and epidural analgesia, three for body temperature ≥ 37.5°C, and five for participants with pregnancy-induced hypertension. The remaining 42 women were enrolled. Two women were dropped out for unilateral block and difficulty with puncture. Finally, a total of 40 women were included in the analysis (Figure 1). The demographics and labor characteristics of maternal subjects are shown in Table 1.

**Table 1:** Demographics and clinical characteristics of maternal participants.

Variables	All (*N* = 40)
Demographics	
Age, year	26.6 ± 3.2
Height, cm	161.2 ± 4.4
Weight, kg	70.2 ± 7.4
Body mass index, kg/m^2^	27.0 ± 2.4
Clinical characteristics	
Gestation, weeks	39.7 ± 0.8
Cervical dilatation at recuitment, cm	2.0 (2.0–2.8)
Baseline HR, bpm	86 ± 13
Baseline SBP, mm Hg	128 ± 10
Baseline DBP, mm Hg	78 ± 8
Baseline Fetal HR, bpm	141 ± 7
Baseline body temperature, °C	36.6 ± 0.3
VAS before analgesia, mm	80 (70–90)

Data are presented as mean ± SD or median (IQR). bpm, beats per minute; DBP, diastolic blood pressure; HR, heart rate; IQR, interquartile range; SBP, systolic blood pressure; SD, standard deviation; VAS, visual analog score.

### 3.2 ED90 and 95% CI of sufentanil

**Figure 2:**
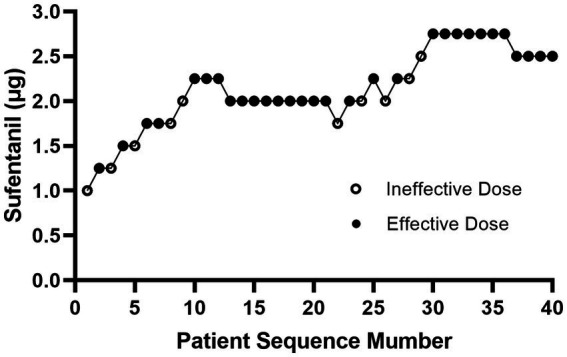
Maternal dose-allocation response sequence.

Figure 2 showed the effective and ineffective responses of 40 consecutive women to different intrathecal doses of sufentanil during labor. The doses ranged from 1.0 to 2.75 μg. Table 2 showed the observed and PAVA-adjusted response rates for each sufentanil dose level. With isotonic regression, the ED90 dose of intrathecal sufentanil combined with ropivacaine 2.5 mg (0.1%, 2.5 mL) was 2.61 μg (95% CI, 2.44–2.70 μg).

**Table 2:** Observed and Pooled Adjacent Violators Algorithm-adjusted response rates.

Assigned dose, μg	No. of patients, N	No. of successes, N	Observed response rate, %	PAVA-adjusted response rate^a^, %	Pruritus, N (%)	Nausea and vomiting, N (%)	Abnormal fetal heart rate, N (%)	Cesarean delivery, N (%)	1 min Apgar score ≤ 7, N (%)
1.00	1	0	0.0	0.0	1 (100.0)	1 (100)	0 (0.0)	0 (0.0)	0 (0.0)
1.25	2	1	50.0	50.0	1 (50.0)	0 (0.0)	0 (0.0)	1 (50.0)	0 (0.0)
1.50	2	1	50.0	50.0	2 (100.0)	0 (0.0)	0 (0.0)	0 (0.0)	0 (0.0)
1.75	4	2	50.0	50.0	3 (75.0)	0 (0.0)	1 (25.0)	1 (25.0)	0 (0.0)
2.00	13	10	76.9	76.9	9 (69.0)	6 (46.0)	2 (15.0)	1 (8.0)	1 (8.0)
2.25	6	5	83.3	81.8	6 (100.0)	3 (50.0)	1 (17.0)	1 (17.0)	1 (17.0)
2.50	5	4	80.0	81.8	3 (60.0)	1 (20.0)	0 (0.0)	2 (40.0)	0 (0.0)
2.75	7	7	100.0	100.0	5 (71.0)	3 (43.0)	1 (14.0)	1 (14.0)	1 (14.0)

PAVA, Pooled Adjacent Violators Algorithm.

^a^ PAVA-Adjusted Response Rates were estimated using the weighted isotonic regression method.

### 3.3 VAS scores at different time points

**Figure 3:**
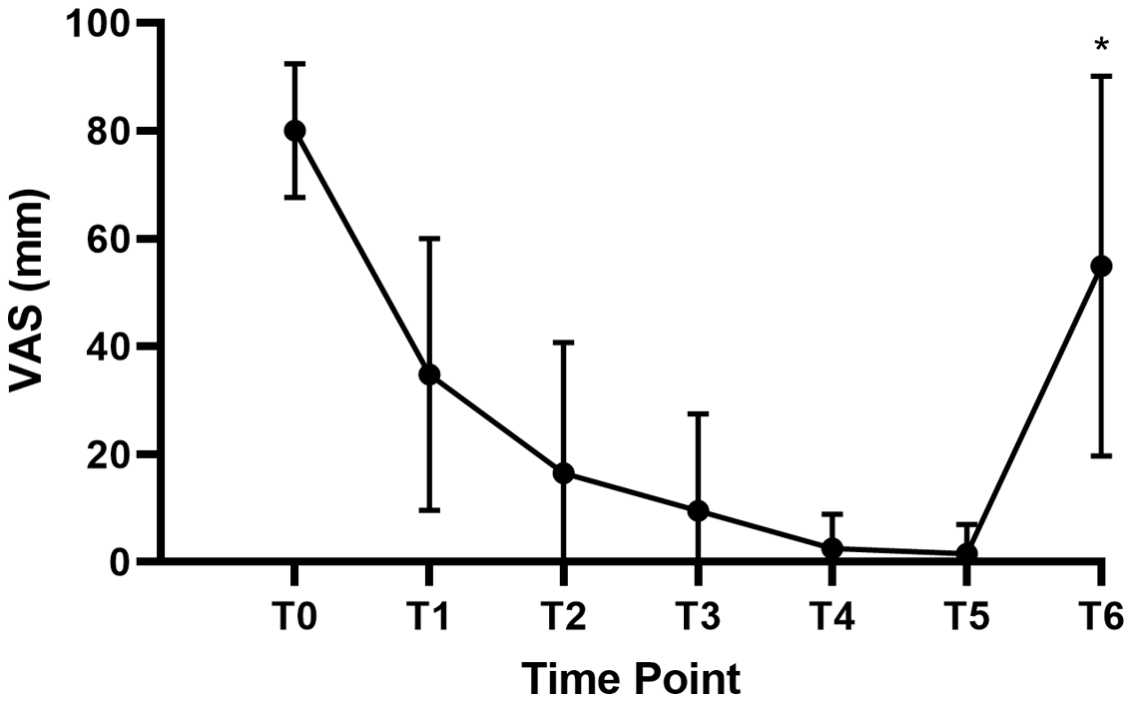
VAS scores of parturients at time points. VAS, visual analog score; T0, Before labor analgesia; T1, 5 min after intrathecal injection; T2, 10 min after intrathecal injection; T3, 15 min after intrathecal injection; T4, 30 min after intrathecal injection; T5, 60 min after intrathecal injection; T6, At the time of full cervical dilation. *T6 only included 33 women with vaginal delivery.

Figure 3 showed the mean VAS scores before labor analgesia (T0), at 5 min (T1), 10 min (T2), 15 min (T3), 30 min (T4), 60 min (T5) after intrathecal injection, and at full cervical dilation (T6).

### 3.4 Maternal outcomes

Table 3 showed the main outcomes of the parturients. Thirty-one (77.5%) parturients complained of pruritus, and 14 (35.0%) suffered nausea and vomiting. Six (15.0%) reported urinary retention, and two (5.0%) were diagnosed with intrapartum fever. No respiratory depression or hypotension was recorded. No women had any degree of motor block. Among all the parturients, 33 had a vaginal delivery and 7 had a cesarean section finally.

**Table 3:** Maternal outcomes.

Variables	All (*N* = 40)
Pruritus	31 (77.5)
Nausea and vomiting	14 (35.0)
Urinary retention	6 (15.0)
Respiratory depression	0 (0.0)
Hypotension	0 (0.0)
Intrapartum fever	2 (5.0)
Abnormal fetal heart rate^a^	5 (12.5)
Modified Bromage score^b^	
0	40 (100.0)
1	0 (0.0)
2	0 (0.0)
3	0 (0.0)
Delivery mode	
Cesarean delivery	7 (17.5)
Vaginal delivery	33 (82.5)
Maternal satisfaction	
Very satisfied	29 (72.5)
Fairly satisfied	11 (27.5)
Not sufficiently satisfied	0 (0.0)
Not at all satisfied	0 (0.0)

Data are presented as N (%).

^a^ Abnormal fetal heart rate (FHR) was defined as late deceleration (FHR < 100 bpm after a contraction) or bradycardia (FHR < 100 bpm for more than 90 s).

^b^ Modified Bromage score (0 = full flexion of knees and ankles, 1 = partial flexion of knees, full flexion of ankles, 2 = inability to flex knees and partial flexion of ankles, and 3 = inability to flex knees and ankles).

### 3.5 Neonatal outcomes

Table 4 showed the primary outcomes of newborns. Three neonates reported a 1 min Apgar score of ≤7, none reported a 5 min Apgar score of ≤7.

**Table 4:** Neonatal outcomes.

Variables	All (*N* = 40)
1 min Apgar score	9 (9–9)
5 min Apgar score	10 (10–10)
1 min Apgar score ≤ 7	3 (7.5)
5 min Apgar score ≤ 7	0 (0.0)

Data are presented as median (IQR) or N (%). IQR, interquartile range.

## 4 Discussion

Our study demonstrated that the optimal dose of intrathecal sufentanil in combination with ropivacaine 2.5 mg to provide effective analgesia for 90% of women was 2.61 (95% CI, 2.44–2.70) μg. The incidence of maternal adverse effects was very low. All newborns were safe.

A fixed intrathecal ropivacaine dose of 2.5 mg was determined based on previous studies. Li et al. (3) injected 5 mL of 0.1% ropivacaine with sufentanil 2.5 μg into the subarachnoid space, reporting the symptoms of warmth and numbness within 3 min were both 100%. However, 77.55% of parturients were found to have a motor block, indicating an overdose of intrathecal drugs. Camorcia et al. (15) found the intrathecal minimum local analgesic dose was 3.64 (95% CI, 3.33 to 3.96) mg for ropivacaine in labor analgesia to achieve an efficacy of VAS score decreased to 10 mm or less within 30 min. Ortner et al. (16) reported the ED 50 of ropivacaine was 4.6 (95% CI, 4.28–5.31) mg when the analgesic effectiveness was defined as a VAS score less than 100 mm at 15 min after intrathecal injection. Adding sufentanil 1.6 and 2.2 μg significantly decreased the ED 50 of ropivacaine to 2.1 mg and 1.9 mg, respectively (16). Given combined sufentanil and ropivacaine for spinal analgesia in our study and the criterion we set for block success was VAS ≤ 30 mm after intrathecal injection at 10 min, we fixed intrathecal ropivacaine dose of 2.5 mg, the same with Levin et al. (17) and Hughes et al. (18) studies.

In our study, 75% of parturients’ VAS score dropped to below 30 mm, and the mean VAS score was 16.5 mm at 10 min after intrathecal injection, indicating a faster onset of analgesia than traditional epidural technique (19, 20) and dural puncture epidural technique (20). No adverse effects, such as hypotension, respiratory depression, motor block, or patient discomfort, were observed after subarachnoid administration.

Compared with epidural labor analgesia, combined spinal-epidural labor analgesia was associated with a higher risk of nonreassuring fetal heart rate (21). A meta-analysis indicated the average incidence of abnormal fetal heart rate was 11.8% in parturients receiving CSE analgesia, which was comparable with our study (5/40, 12.5%). Among the five cases in our study, four recovered in a very short period of time, and one performed an emergency cesarean section due to fetal bradycardia. The overall cesarean section rate was basically consistent with previous reports (22, 23). From the results in Table 2, we have not found any close relationship between the incidences of abnormal fetal heart and cesarean section and the dose of sufentanil.

Intrathecal injection of opioids is the main culprit causing pruritus. Our study showed 77.5% of participants had suffered pruritus, similar to previously reported studies (24, 25). However, most symptoms were mild and transient and did not require pharmacological treatment. Herman et al. reported the incidence of pruritus in parturients receiving intrathecal opioids during labor displayed a dose–response in relationship identical to that seen for analgesia (26). However, no dose–response relationship was found in our study. It may be due to a small sample size of each group. The incidence of nausea and vomiting in our study was 35%, similar to previously reported studies (27).

There are some limitations in our study. Firstly, our results may not be applicable to either multiparous women or nulliparous women in advanced labor. Secondly, the ED90 of sufentanil observed in our study may only be valid for combining with ropivacaine 2.5 mg, since there is a pharmacologic synergistic interaction between intrathecal opioid and local anesthetic given intrathecally for labor analgesia (28). Thirdly, we did not measure the maternal sensory block level. However, no parturient developed respiratory depression in our study indicating no high block level occurred. We will include maternal sensory block level assessment in further study.

## 5 Conclusion

The ED90 of intrathecal sufentanil combined with ropivacaine 2.5 mg for labor analgesia was 2.61 (95% CI, 2.44 to 2.70) μg. The dose is safe for parturients and neonates.

## Data availability statement

The raw data supporting the conclusions of this article will be made available by the authors, without undue reservation.

## Ethics statement

The studies involving humans were approved by Research Ethics Committee at Peking University First Hospital Ningxia Women’s and Children’s Hospital in Yinchuan, China (KJ-LL-2021-42, approval date November 25, 2021). The studies were conducted in accordance with the local legislation and institutional requirements. The participants provided their written informed consent to participate in this study. Written informed consent was obtained from the individual(s) for the publication of any potentially identifiable images or data included in this article.

## Author contributions

QY: Conceptualization, Methodology, Project administration, Writing – review & editing. BY: Conceptualization, Methodology, Writing – review & editing. HH: Methodology, Writing – original draft. GL: Formal analysis, Writing – review & editing. JS: Writing – review & editing. HK: Conceptualization, Methodology, Project administration, Writing – original draft. LD: Conceptualization, Methodology, Project administration, Writing – review & editing.
